# A review of traditional pharmacological uses, phytochemistry, and pharmacological activities of *Tribulus terrestris*

**DOI:** 10.1186/s13065-017-0289-x

**Published:** 2017-07-11

**Authors:** Wenyi Zhu, Yijie Du, Hong Meng, Yinmao Dong, Li Li

**Affiliations:** School of Science, Beijing Technology and Business University, Beijing, 100048 People’s Republic of China

**Keywords:** *Tribulus terrestris*, Traditional uses, Phytochemical, Pharmacological activities

## Abstract

*Tribulus terrestris* L. (TT) is an annual plant of the family Zygophyllaceae that has been used for generations to energize, vitalize, and improve sexual function and physical performance in men. The fruits and roots of TT have been used as a folk medicine for thousands of years in China, India, Sudan, and Pakistan. Numerous bioactive phytochemicals, such as saponins and flavonoids, have been isolated and identified from TT that are responsible alone or in combination for various pharmacological activities. This review provides a comprehensive overview of the traditional applications, phytochemistry, pharmacology and overuse of TT and provides evidence for better medicinal usage of TT.

## Introduction

TT is an annual plant of the family Zygophyllaceae, which is commonly known as *Tribulus*, Hard thorns, and goat head in China. It is mainly planted in the Mediterranean and in sub-tropical regions such as India, China, South America, Mexico, Spain, Bulgaria, and Pakistan. It is a small, prostrate, 10–60 cm high, hirsute or silky hairy shrub. The leaves are opposite, often unequal, paripinnate, pinnate from 5 to 8 pairs and elliptical or an oblong lanceolate. The fruits from the five mericarps are ax-shaped, 3–6 mm long, and arranged radially and have a diameter of 7–12 mm and a hard texture. The root is slender, fibrous, cylindrical and frequently branched, bears a number of small rootlets and is light brown in colour [1]. The fruits and roots of TT, as a folk medicine, have been used for thousands of years in China. Over the last several years, it has been certified for its pharmaceutical activities for improving sexual function and cardiac protection and providing anti-urolithic, antidiabetic, anti-inflammatory, antitumour and antioxidants effects.

In the current review, we present and analyse the ethnobotanical use and the phytochemical and pharmacological activities of TT. These up-to-date research observations will be helpful in understanding the characteristics and superiorities of this traditional Chinese medicine and will be applicable in developing new products and herbal medicines in the future.

## Traditional pharmacological uses

TT is native to south-eastern and Mediterranean Europe, temperate and tropical Asia and Africa, and northern Australia. The use of TT from ancient times occurred in the traditional medicine of major cultures in these geographical areas, such as traditional Chinese medicine, traditional Indian medicine (Ayurveda), and the traditional medicine of south-eastern Europe, and this has defined its ethnopharmacological relevance as a medicinal plant [2]. As a traditional Chinese Medicine, it was listed as a top grade medicine in the earliest extant Chinese pharmaceutical monograph “Shen Nong Ben Cao Jing” [3]. In Chinese Pharmacopoeia [4], the fruits of TT have been used for tonifying the kidneys and as a diuretic and cough expectorant that improves eyesight and for the treatment of skin pruritus, headache and vertigo, and mammary duct blockage. In India, the fruits have been used in the treatment of infertility, impotence, erectile dysfunction and low libido in Ayurveda. In addition, the roots and fruits are considered to have cardiotonic properties [5]. In Sudan, TT has been used as demulcent and in nephritis and the treatment of inflammatory disorders [5]. In addition, it has been used for diuretic and uricosuric effects in Pakistan [6]. Modern investigation showed that the chemical constituents steroidal saponins and flavonoids with the prominent anti-inflammatory and antiaging activities of TT were the main contributors to the traditional pharmacological activities.

## Phytochemical investigations

Many different compounds with a variety of biological properties and chemical structures have been identified from TT, including steroidal saponins, flavonoids, glycosides, phytosterols, tannins, terpenoids, amide derivatives, amino acids, and proteins. Among the different types of constituents, steroidal saponins and flavonoids are considered to be the most important metabolites with various bioactivities.

### Steroidal saponins

**Fig. 1:**
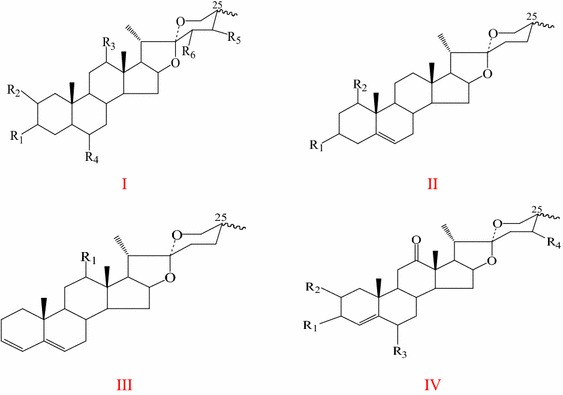
Skeletal types of spirostane saponins in *T. terrestris*

**Fig. 2:**
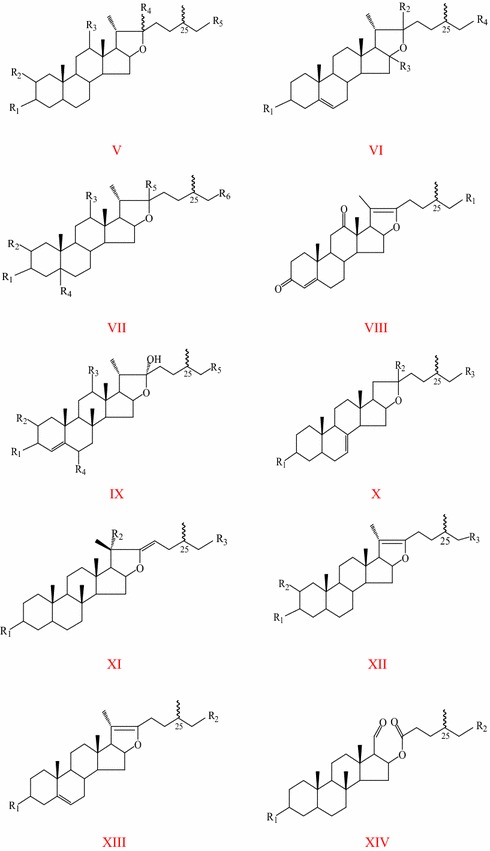
Skeletal types of furostane saponins in *T. terrestris*

Spirostanol and furostanol saponins are considered the most characteristic chemicals in TT. To date, 108 kinds of steroidal saponins have been isolated from TT **(1–108)**. Among them, there are 58 kinds of spirostane saponins **(1–58)** and 50 kinds of furostane saponins **(59–108)**. The steroidal saponins, such as protodioscin and protogracillin, are thought to confer TT unique biological activities. Skeletal types of steroidal aglycones in TT are shown in Figs. 1, 2. Steroidal saponins(aglycones) in TT are shown in Table 1.

**Table 1:** Steroidal saponins (aglycones) in *T. terrestris*

No.	Chemicals	Aglycones	R_1_	R_2_	R_3_	R_4_	R_5_	R_6_	25-C	Refs.
1	Tigogenin	I	OH	H	H	H	H	H	R	[7]
2	Neotigogenin	I	OH	H	H	H	H	H	S	[8]
3	Tigogenin-3-*O*-*β* -d-glc(1 → 4)-*β* -d-gal (A)	I	Ra	H	H	H	H	H	R	[9]
4	Terrestrosin F	I	Rb	H	H	H	H	H	R	[10]
5	Desgalactotigonin	I	Rc	H	H	H	H	H	R	[9, 11]
6	Gitonin (B)	I	Rd	H	H	H	H	H	R	[11]
7	Tigogenin-3-*O*-*β* -d-xyl-(1 → 2)-[*β* -d-xyl-(1 → 3)]-*β* -d-glc(1 → 4)-[*α* -l-rha(1 → 2)]-*β* -d-gal (C)	I	Re	H	H	H	H	H	R	[9, 11, 12]
8	Tribulosin	I	Re	H	H	H	H	H	S	[13]
9	Terrestrosin A	I	Rf	H	H	H	H	H	R,S	[11]
10	Terrestrosin B	I	Rg	H	H	H	H	H	R,S	[11]
11	Gitogenin	I	OH	OH	H	H	H	H	R	[7, 14]
12	Neogitogenin	I	OH	OH	H	H	H	H	S	[15]
13	Gitogenin-3-*O*-*β* -d-glc(1 → 4)-*β* -d-gal	I	Ra	OH	H	H	H	H	R	[16]
14	F-gitonin	I	Rd	OH	H	H	H	H	R	[11]
15	Desglccolanatigonin	I	Rd	OH	H	H	H	H	R	[11]
16	25R,S-5*α*-spiro-2*α*,3*β*-dihydroxyl-3-*O*-*β* -d-glc-(1 → 4)-*β* -d-gal	I	Ra	OH	H	H	H	H	R,S	[16]
17	Terrestrosin E	I	Rf	OH	H	H	H	H	R,S	[11, 16]
18	Hecogenin	I	OH	H	=O	H	H	H	R	[7, 17]
19	Neohecogenin sapogenin	I	OH	H	=O	H	H	H	S	[17]
20	Agovoside A	I	Rh	H	=O	H	H	H	R	[12]
21	Hecogenin-3-*O*-*β* -d-glc-(1 → 4)-*β* -d-gal (D)	I	Ra	H	=O	H	H	H	R	[9, 18]
22	Hecogenin-3-*O*-*β* -d-glc-(1 → 2)-*β* -d-glc(1 → 4)-*β* -d-gal (E)	I	Ri	H	=O	H	H	H	R	[9]
23	Hecogenin-3-*O*-*β* -d-xyl-(1 → 3)-*β* -d-glc(1 → 4)-*β* -d-gal (F)	I	Rj	H	=O	H	H	H	R	[9]
24	Hecogenin-3-*O*-*β* -d-glc-(1 → 2)-[3-*O*-*β* -d-xyl]-*β* -d-glc-(1 → 4)-*β* -d-gal (G)	I	Rd	H	=O	H	H	H	R	[9, 12]
25	Terrestrosin D	I	Rk	H	=O	H	H	H	R	[11, 12]
26	Hecogenin-3-*O*-*β* -d-xyl-(1 → 2)-[*β* -d-xyl-(1 → 3)]-*β* -d-glc(1 → 4)-[*α* -l-rha-(1 → 2)]-*β* -d-gal (H)	I	Re	H	=O	H	H	H	R	[12]
27	Neohecogenin-3-*O*-*β* -d-glc	I	Rl	H	=O	H	H	H	S	[8]
28	Terreside B	I	Ra	H	=O	H	H	H	S	[19]
29	Terreside A	I	Ri	H	=O	H	H	H	R	[19]
30	Tettestrosin C	I	Rf	H	=O	H	H	H	R,S	[11, 13]
31	25R,S-5*α*-spiro-12-one-3-*O*-*β* -d-xyl-(1 → 2)-[*β* -d-xyl-(1 → 3)]-*β* -d-glc(1 → 4)-[*α* -l-rha(1 → 2)]-*β* -d-gal	I	Re	H	=O	H	H	H	R,S	[20]
32	Hecogenone	I	=O	H	=O	H	H	H	R	[7]
33	25R-5*α*-spiro-3,6,12-triketo	I	=O	H	=O	=O	H	H	R	[7]
34	Sarsasaponin	I	OH	H	H	H	H	H	S	[21]
35	Isoterrestrosin B(I)	I	Rg	H	H	H	H	H	S	[21]
36	Chlorogenin	I	OH	H	H	OH	H	H	R	[22]
37	Terrestrinin U	I	H	Rm	H_2_	H	Rl	H	S	[23]
38	Terrestrinin I	I	Ra	H	=O	H	Rl	H	S	[24]
39	23S,25S-5*α*-spiro-24-one-3*β*,23-diol-3-*O*-[*α* -l-rha-(1 → 2)]-*O*-[*β* -d-glc-(1 → 4)]-*β* -d-gal	I	Rn	H	H	H	=O	OH	S	[25]
40	24S,25S-5*α*-spiro-3*β*,24-diol-3-*O*-[*α* -l-rha-(1 → 2)]-*O*-[*β* -d-glc-(1 → 4)]-*β* -d-gal	I	Rn	H	H	H	OH	H	S	[25]
41	23S,24R,25R-5*α*-spiro-3*β*,23,24-triol-3-*O*-[*α* -l-rha-(1 → 2)]-[*β* -d-glc-(1 → 4)]-*β* -d-gal	I	Rn	H	H	H	OH	OH	R	[26]
42	23S,24R,25S-5*α*-spiroe-3*β*,23,24-triol-3-*O*-[*α* -l-rha-(1 → 2)-[*β* -d-glc-(1 → 4)]-*β* -d-gal	I	Rn	H	H	H	OH	OH	S	[26]
43	Diosgenin	II	OH	H					R	[14]
44	Yamogenin	II	OH	H					S	[10]
45	Trillin	II	Rl	H					R	[27]
46	Trillarin	II	Ro	H					R	[27]
47	Dioscin	II	Rp	H					R	[28]
48	Gracillin	II	Rq	H					R	[27]
49	Diosgenin-3-*O*-*α* -l-rha-(1 → 3)-*β* -d-glc	II	Rr	H					R	[27]
50	Tribestin	II	Rs	H					R	[28]
51	Ruscogenin	II	OH	OH					R	[14]
52	Saponin C	II	OH	Rt					R	[29]
53	25R-spiro-3,5-diene	III	H						R	[14]
54	25R-spiro-3,5-dien-12one	III	=O						R	[17]
55	25R-spiro-4-ene-3,12-dione	IV	=O	H	H	H			R	[7, 17]
56	25R-spiro-4-ene-3,6,12-trione	IV	=O	H	=O	H			R	[7]
57	25R-spiro-24-hydroxy-3,12-dione	IV	=O	H	H	OH			R	[17]
58	25R-spiro-2*α*,3*β*-hydroxy-4-ene-12one	IV	OH	OH	H	H			R	[17]
59	25R-5*α*-furo-22-methoxy-3*β*,26-dihydroxy-*O*-*β* -d-glc-3-*O*-*β* -d-xyl-(1 → 2)-[β-d-xyl-(1 → 3)]-*β* -d-glc(1 → 4)-[*α* -l-rha(1 → 2)]-*β* -d-gal (J)	V	Re	H	H	OCH_3_	Rl		R	[9, 12, 21]
60	Neoprotodioscin	V	Rp	H	H	OH	Rl		R	[29]
61	Neoprototribestin	V	Rs	H	H	OH	Rl		R	[24]
62	Terrestrinin B	V	Re	H	H	OH	Rl		S	[12]
63	Tettestrosin H	V	Rf	H	H	OH	Rl		R,S	[16]
64	Terrestroneoside A	V	Re	H	H	OCH_3_	Rl		Uncertain	[30]
65	Terrestrosin F	V	Ra	OH	H	OH	Rl		R	[16]
66	25R,S-5*α*-furo-2*α*,3*β*,22*α*,26-tetrahydroxy-*O*-*β* -d-glc-(1 → 4)-*β* -d-gal	V	Ra	OH	H	OH	Rl		R,S	[16]
67	Terrestrosin G	V	Rf	OH	H_2_	OH	Rl		R,S	[16]
68	25R,5*α*-furo-12-one-3*β*,22*α*,26-trihydroxy-*O*-*β* -d-glc-3-*O*-*β* -d-glc-(1 → 2)-*β* -d-gal	V	Ru	H	=O	OH	Rl		R	[31]
69	25S,5*α*-furo-12-one-3*β*,22*α*,26-trihydroxy-*O*-*β* -d-glc-3-*O*-*β* -d-glc-(1 → 2)-*β* -d-gal	V	Ru	H	=O	OH	Rl		S	[20]
70	25S,5*α*-furo-12-one-3*β*,22*α*,26-trihydroxy-*O*-*β* -d-glc-3-*O*-*β* -d-glc-(1 → 4)-[*α* -l-rha-(1 → 2)]-*β* -d-gal	V	Rg	H	=O	OH	Rl		S	[20]
71	Terrestrosin I	V	Rf	H	=O	OH	Rl		R,S	[16]
72	5*α*-furo-12-one-3*β*,22,26-trihydroxy-*O*-*β* -d-glc-3-*O*-*β* -d-xyl-(1 → 3)-[*β* -d-gal-(1 → 2)]-*β* -d-glc(1 → 4)-*β* -d-glc	V	Rv	H	=O	OH	Rl		Uncertain	[18]
73	Terrestrinin R	V	H	Rm	=O	OH	Rl		R	[23]
74	Terrestrinin S	V	H	Rm	=O	OH	Rl		S	[23]
75	Terrestrinin F	V	OH	H	=O	OH	Rl		R	[24]
76	26-*O*-*β* -d-glc-25R-5*α*-furo-2*α*,3*β*,22*α*,26-tetraol-3-*O*-*β* -d-glc-(1 → 2)-*O*-*β* -d-glc-(1 → 4)-*β* -d-gal	V	Ri	OH	H	CH_3_	Rl		R	[25]
77	Pseudoprotodioscin(K)	VI	Rp	OH	H	Rl			R	[21]
78	Methyl pseudo-diosgenin	VI	Rp	OCH_3_	H	Rl			R	[32]
79	Prototribestin	VI	Rs	OH	H	Rl			R	[32]
80	Methyprototribestin	VI	Rs	OCH_3_	H	Rl			R	[32]
81	Protogracillin	VI	Rq	OH	H	Rl			R	[8]
82	Terrestrosin J	VI	Rf	OH	H	Rl			R,S	[16]
83	Tribol	VI	Rp	H	OH	H			R	[28]
84	Terrestrinin K	VI	Rm	OH	H	Rl			R	[23]
85	Terrestrosin K	VII	Rf	H	=O	H	CH_3_		R	[16]
86	25R,S-5*α*-furo-12-one-20,22-ene-3*β*,26-dihydroxy-*O*-*β* -d-glc-3-*O*-*β* -d-glc-(1 → 4)-*β* -d-gal	VII	Ra	H	=O	H	CH_3_	Rl	R,S	[31]
87	25R,S-5*α*-furo-12-one-20,22-ene-3*β*,26-dihydroxy-*O*-*β* -d-gal-(1 → 2)-*β* -d-glc-(1 → 4)-*β* -d-gal	VII	Rf	H	=O	H_2_	CH_3_	Rl	R,S	[16]
88	5*α*-furo-12-one-20,22-ene-3*β*,26-dihydroxy-*O*-*β* -d-glc-3-*O*-*β* -d-xyl-(1 → 3)-[*β* -d-gal-(1 → 2)]-*β* -d-glc-(1 → 4)-*β* -d-glc	VII	Rv	H	=O	H_2_	CH_3_	Rl	Uncertain	[18]
89	Tribulosaponin A(L)	VII	Rp	H	H	H_2_	CH_3_	Rl	S	[21]
90	Tribulosaponin B(M)	VII	Rg	H	H	H_2_	CH_3_	Rl	S	[21]
91	26-*O*-*β* -d-glc-25R-5*α*-furo-20(22)-en-2*α*,3*β*,26-triol-3-*O*-*β* -d-glc-(1 → 2)-*O*-*β*-glc-(1 → 4)-*β* -d-gal	VII	Rn	H	=O	H	OCH_3_	Rl	R	[25]
92	26-*O*-*β* -d-glc-25R-5*α*-furo-12-one-3*β*,22*α*,26-triol-3-*O*-*β* -d-glc-(1 → 2)-*β* -d-glc-(1 → 4)-*β* -d-gal	VII	Ri	H	=O	H	OH	Rl	R	[33]
93	26-*O*-*β* -d-glc-25S-5*α*-furo-22-methoxy-2*α*,3*β*, 26-triol-3-*O*-*β* -d-glc-(1 → 2)-*β* -d-glc-(1 → 4)-*β* -d-gal	VII	Ri	OH	H	H	OCH_3_	Rl	S	[33]
94	Terrestrinin A	VIII	Rl						S	[12]
95	Terrestrinin Q	VIII	Rl						R	[23]
96	Terrestrinin C	IX	OH	OH	=O	H_2_	Rl		R	[24]
97	Terrestrinin D	IX	=O	H	=O	H_2_	Rl		R	[24]
98	Terrestrinin E	IX	=O	H	=O	=O	Rl		R	[24]
99	Terrestrinin G	IX	=O	H	OH	H_2_	Rl		R	[24]
100	Terrestrinin H	IX	=O	H	=O	H_2_	Rv		R	[24]
101	Terrestrinin J	X	Rm	OH	Rl				R	[23]
102	Terrestrinin L	XI	Rm	OH	Rl				R	[23]
103	Terrestrinin M	XI	Rx	OH	Rl				R	[23]
104	Terrestrinin N	XI	Ry	OH	Rl				R	[23]
105	Terrestrinin O	XII	Ry	H	Rl				R	[23]
106	26-*O*-*β* -d-glc-25R-5*α*-furo-20(22)-en-2*α*,3*β*, 26-triol-3-*O*-*β* -d-glc-(1 → 2)-*O*-*β* -d-glc-(1 → 4)-*β* -d-gal	XII	Ri	OH	Rl				R	[25]
107	Terrestrinin P	XIII	Rm	Rl					R	[23]
108	Terrestrinin T	XIV	Rm	Rl					R	[23]

Ra: *O*-*β* -d-Gal-(1 → 4)-*β* -d-Glc; Rb: *O*-Glc/Rha = 2:1; Rc: *O*-*β* -d-Gal-[(1 → 2)-*β* -d-Xyl]-(1 → 4)-*β* -d-Glc-(1 → 2)-*β* -d-Glc; Rd: *O*-*β* -d-Gal-(1 → 4)-*β* -d-Glc-[(1 → 3)-*β* -d-Xyl]-(1 → 2)-*β*-D—Glc; Re: *O*-*β* -d-Gal-[(1 → 2)-*α* -l-Rha]-(1 → 4)-*β* -d-Glc-[(1 → 3)-*β* -d-Xyl-(1 → 2)-*β*-Xyl; Rf: *O*-*β* -d-Gal-(1 → 4)-*β* -d-Glc-(1 → 2)-*β* -d-Gal; Rg: *O*-*β* -d-Gal-[(1 → 2)-*α* -l-Rha]-(1 → 4)-*β* -d-Glc; Rh: *O*-*β* -d-Gal; Ri: *O*-*β*-Gal-(1 → 4)-*β*-Glc-(1 → 2)-*β* -d-Glc; Rj: *O*-*β* -d-Gal-(1 → 4)-*β* -d-Glc-(1 → 3)-*β* -d-Xyl; Rk: *O*-*β* -d-Gal-(1 → 4)-*β* -d-Glc-[(1 → 2)-*β* -d-Gal]-(1 → 3)-*β* -d-Xyl; Rl: *O*-*β* -d-Glc; Rm: *O*-*β* -d-Gal-[(1 → 2)-*α* -l-Rha]-(1 → 4)-*β* -d-Glc-[(1 → 3)-*β* -d-Xyl]-(1 → 2)-*β* -d-Xyl; Rn: *O*-*β* -d-Gal-[(1 → 4)-*β* -d-Glc]–[(1 → 2)-*α* -l-Rha]; Ro: *O*-*β* -d-Glc-(1 → 2)-*β* -d-Glc; Rp: *O*-*β* -d-Glc-[(1 → 2)-*α* -l-Rha]-(1 → 4)-*α* -l-Rha; Rq: *O*-*β* -d-Glc-[(1 → 2)-*α* -l-Rha]-(1 → 3)-*β* -d-Glc; Rr: *O*-*β* -d-Glc-(1 → 3)-*α* -l-Rha; Rs: *O*-*β* -d-Glc-[(1 → 2)-*α* -l-Rha]-(1 → 4)-SO_3_Na; Rt: *O*-*β* -d-Glc-[(1 → 2)-*α* -l-Rha]-(1 → 6)-OAc; Ru: *O*-*β* -d-Gal-(1 → 2)-*β* -d-Glc; Rv: *O*-*β* -d-Glc-(1 → 4)-*β* -d-Glc-[(1 → 2)-*β* -d-Gal]-(1 → 3)-*β* -d-Xyl; Rw: *O*-*β* -d-Glc-(1 → 6)-*β* -d-G; Rx: *O*-*β* -d-Gal-(1 → 4)-*β* -d-Glc-[(1 → 3)-*β* -d-Xyl]-(1 → 2)-*β* -d-A; Ry: *O*-*β* -d-Gal-(1 → 4)-*β* -d-Glc-[(1 → 3)-*β* -d-Xyl]-(1 → 2)-*β* -d-Xyl

### Flavonoids

**Fig. 3:**
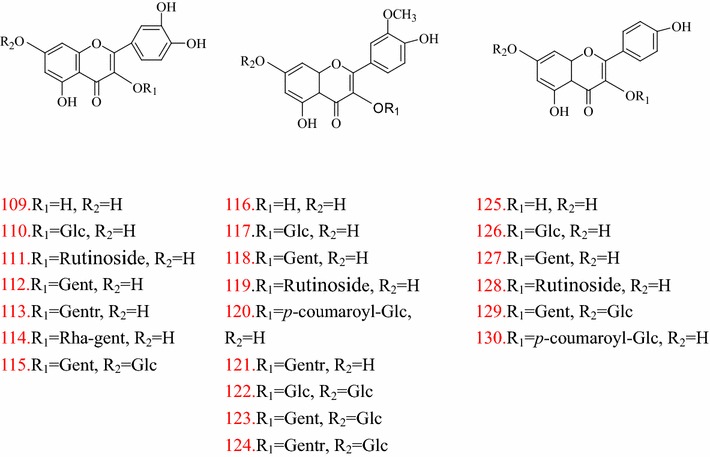
Structures of flavonoids in *T. terrestris*

The flavonoids of TT are mainly derivatives of quercetin, kaempferol and isorhamnetin. Quercetin **(109)**, isoquercitrin** (110)**, rutin **(111)**, quercetin-3-*O*-gent **(112)**, quercetin-3-*O*-gentr** (113)**, quercetin-3-*O*-rha-gent **(114)**, quercetin-3-*O*-gent-7-*O*-glu **(115)** are flavonoids with quercetin as the basic parent structure [34–36]. Isorhamnetin **(116)**, isorhamnetin-3-*O*-glu **(117)**, isorhamnetin-3-*O*-gent **(118)**, isorhamnetin-3-*O*-rutinoside **(119)**, isorhamnetin-3-*O*-gentr **(120)**, isorhamnetin-3,7-di-*O*-glu **(121)**, isorhamnetin-3-*O*-*p*-coumarylglu** (122)**, isorhamnetin-3-*O*-gent-7-*O*-glu** (123)**, isorhamnetin-3-*O*-gentr-7-*O*-glu** (124)** are flavonoids with isorhamnetin as the basic parent structure [30, 32, 37]. Kaempferol **(125)**, kaempferol-3-*O*-glu **(126)**, kaempferol-3-*O*-gent **(127)**, kaempferol-3-*O*-rutinoside **(128)**, kaempferol-3-*O*-gent-7-*O*-glu** (129)**, tribuloside **(130)** are flavonoids with kaempferol as the basic parent structure [35, 36, 38, 39]. Structures of flavonoids in TT are shown in Fig. 3.

### Alkaloids

**Fig. 4:**
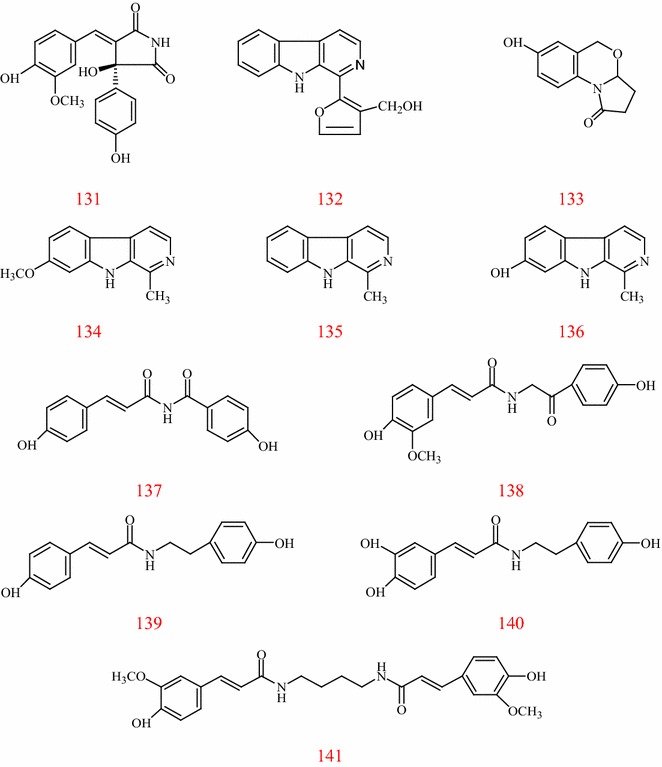
Structures of alkaloid in *T. terrestris*

Tribulusamide C **(131)**, tribulusterine **(132)**, tribulusin A **(133)**, harmine** (134)**, harman **(135)**, harmmol **(136)**, tribulusimide C** (137)**, terrestriamide **(138)**, *N*-*trans*-coumaroyltyramine **(139)**, *N*-*trans*-caffeoylyramine **(140)**, terrestribisamide** (141)** are the main alkaloids isolated from the stems, leaves, and fruits of TT [40–45]. The nuclear mainly belong to *β*-carboline alkaloids and amide alkaloids. Structures of the alkaloids in TT are shown in Fig. 4.

### Others

Other components of TT include organic acids, amino acids and other substances. Organic acids isolated from TT are benzoic acid [46], vanillic acid, 2-methyl benzoic acid, ferulic acid [42], succinic acid, palmitic acid monoglyceride, succinic acid, docosanoic acid [47], Tribulus acid [48] and others. The main amino acids are alanine and threonine [49]. In addition, TT also contains 4-ketopinoresinol [50], uracil nucleic acid [46], coumarin [47], emodin, and physcion [51].

## Pharmacological activities

TT has long been used in traditional Chinese and Indian systems of medicine for the treatment of various ailments, especially for improving sexual function, the prevention and treatment of cardiovascular diseases, and diabetes. It also has hepatoprotective, antioxidant, anti-inflammatory, antibacterial, antiaging, and antitumour activities.

### Improving sexual function

The active extracts and constituents of TT could improve sexual function through activating aphrodisiacs and improving fertility in men. It could also activate sexual desire in postmenopausal women. It is widely believed and insistently advertised that TT possesses aphrodisiac and pro-sexual activities due to its ability to increase testosterone or testosterone precursor levels and this view is outdated [2].

### Aphrodisiac activation

Erectile dysfunction (ED) is a sexual disorder characterized by the inability to achieve or maintain a sufficiently rigid erection [52]. Analysis of phytochemical and pharmacological studies in humans and animals revealed an important role for *T. terrestris* in treating erectile dysfunction and sexual desire problems. Rats were fed a standard diet treated with Mucuna pruriens, *T. terrestris*, and Ashwagandha (300 mg kg^−1^) for 8 weeks. The results indicated that the extract of TT was comparatively more potent than the two others. These herbs are potent enhancers of sexual function and behaviour by increasing the testosterone levels and regulating the NF-κB and Nrf2/HO–1 pathways in male rats [53].

The hormonal effects of TT were evaluated in primates, rabbits and rats to identify its usefulness in the management of ED [54]. Blood samples were analysed for testosterone (T), dihydrotestosterone (DHT) and dehydroepiandrosterone sulphate (DHEAS) levels using a radioimmunoassay. TT increased some of the sex hormones, which is possibly due to the presence of protodioscin in the extract. The results indicated that TT may be useful in mild to moderate cases of ED.

The aphrodisiac properties of the furostenol glycoside fraction of *T. terrestris* extract (TT-FG) were previously studied [55]. Adult Wister rats were castrated and divided into five groups of six animals each and treated with TT-FG (5, 10, and 25 mg kg^−1^, p.o.) once daily through subcutaneous injections for 14 days. After the acute (1 day) and subacute (7 and 14 days) treatments with the TT-FG, there was an increase in mounting frequency (MF), intromission frequency (IF), and ejaculation latency (EL) and a decrease in mounting latency (ML), intromission latency (IL), and post-ejaculation interval (PEI) and serum testosterone levels in the blood.

There was a randomized, double-blind, placebocontrolled, clinical trial as a piece of evidence for aphrodisiac activation function of TT. 180 males aged between 18 and 65 years with mild or moderate ED and with or without Hypoactive sexual desire disorder (HSDD) were randomized in a 1:1 ratio to the two treatments groups (TT or placebo). The TT group received 2 tablets (500 mg) Tribestan orally three times daily after meals for 12 weeks. Each tablet contains the active substance TT herba extractum siccum 250 mg (content of furostanol saponins not less than 112.5 mg). And the placebo group were treated by a identical appearance, colour and taste one. The results showed that there was significant differences of IIEF (International Index of Erectile Function) score between the two groups (p < 0.0001) after 3 months, but no differences in the incidence of adverse effects [56]. It can therefore be assumed that TT can improve sexual function.

### Improvement in fertility

In the literature, it has been concluded that the ethanol extract of *T. terrestris* (EETT) influences spermatogenesis, as shown by the evident changes in the tubular compartment of the testes, such as increases in the total tube length, tubular volume and height of the seminiferous epithelium. The hexanic and aqueous soluble fraction in the methanol fractions promoted changes in the intertubular compartment because they increased the nuclear volume, cytoplasmic volume and individual volume of Leydig cells in male Wistar rats [57].

Another animal study describes the protective role of TT against AlCl_3_-induced adverse effects on male reproductive organs and fertility. High dosages of TT (100 mg kg^−1^ day^−1^) in AlCl_3_-treated mice restored the body weight, sex organ relative weights, sperm count, motility, viability, epididymal sialic acid, seminal vesicular fructose, serum testosterone, antioxidant enzymes [superoxide dismutase (SOD), catalase (CAT), and glutathione peroxidase-1 (GPx)], mating ability and fertility [58].

TT was reported to cause reproductive system enhancement and possess antioxidant activity, which may assist in the choice of drugs for longer durations that can be prescribed safely without affecting the fertility potential in males. A high dose of the fruit extract of TT (200 mg kg^−1^ day^−1^) restored metronidazole (MTZ)-induced spermatogenic inhibition and reduced the epididymal sperm count. The restoring potential of TT against MTZ-induced alterations in the spermatogenesis appears to be due to the presence of antioxidative flavonoids rather than steroidal saponins [59].

The in vitro addition of TT extract to human sperm could affect male fertility capacity. The incubation of human semen with 40 and 50 μg mL^−1^ of TT extract significantly enhanced the total sperm motility, number of progressive motile spermatozoa, and curvilinear velocity over 60–120 min of holding time. Overall, the sperm viability significantly improved [60].

### Libido-enhancing activity

HSDD is defined in Diagnostic and Statistical Manual of Fourth Edition as persistent or recurrent deficiency (or absence) of sexual fantasies/thoughts, and/or desire for or receptivity to sexual activity, which causes personal distress [61]. TT was considered to be a safe alternative for the treatment of HSDD in postmenopausal women because it was effective in reducing symptoms with few side effects through a randomized, double-blinded, placebo-controlled trial (A total of 45 healthy sexually active postmenopausal women who reported a diminished libido were selected to participate in the study and were randomly assigned to receive 750 mg day^−1^ of TT or a placebo for 120 days). Its probable mechanism of action involves an increase in the serum levels of free and bioavailable testosterone [62].

Other clinical research established that regarding the treatment in the domains of desire and sexual interest of 74 postmenopausal women with sexual dysfunction, the TT treatment (250 mg, orally three times a day for 90 days) was considered to be effective in treating sexual problems among menopausal women [63].

#### Antiurolithic activity

The fruits of TT have long been used in traditional systems of medicine for the treatment of various urinary diseases including urolithiasis. Calcium oxalate is a major type of crystal found in kidney stones. Calcium oxalate is classified into two types: calcium oxalate monohydrate stones (COM) and calcium oxalate dihydrate (COD). Many medicinal plants have been used for centuries for the treatment of urinary stones in spite of the lack of rationale behind their use. The aqueous extract of TT fruits and its fractions were studied to evaluate its antiurolithiatic potential using different models. The inhibitory potency of the plant was tested on the nucleation and growth of the most commonly occurring kidney stones and COM. The results showed that the bioactive *n*-butanol fraction, due to higher contents of quercetin, diosgenin and tannic acid, has a protective capacity rather than a curative property against urolithiasis [64].

A protein (60 kDa) purified from TT showed the highest similarity with carotenoid cleavage dioxygenase 7 (CCD_7_) of Arabidopsis thaliana after matching peptide mass fingerprints with the MASCOT search engine. CCD_7_ belong to a family of dioxygenases, which possess five characteristic conserved histidines spread throughout their primary protein sequence. Histidine is said to induce the conversion of oxalate to formic acid and carbon dioxide (CO_2_). The purified protein decreased cell injury induced by oxalate in a concentration dependent manner and showed the ability to inhibit calcium oxalate (CaOx) crystallization in vitro [65].

Human clinical data indicated that TT extract may be useful in the treatment of urolithiasis. After oral administration of the extract, the levels of mean citrate, oxalate, proteins and glycosaminoglycan in patients’ 24 h urine samples decreased significantly. Urine volume and phosphate level in the serum were not altered significantly in the urolithic patients [66]. It was concluded that TT extract was useful in the treatment of urolithiasis.

#### Antidiabetic activity

Diabetes mellitus is a metabolic disorder with chronic hyperglycaemia, which results from a defect in insulin secretion, insulin action, or both [67]. The gross saponins of *T. terrestris* (GSTT) showed inhibitory activity against *α*-glucosidase. In addition, it showed the inhibition activities of a postprandial increase in blood glucose and improvement in insulin dependent diabetes symptoms [68]. Animal experiments indicated that GSTT significantly reduced the postprandial blood glucose levels by intragastric administration of sucrose in normal rats and type 2 diabetic rats but did not affect the postprandial blood glucose levels of the rats with intragastric administration of glucose [69]. Clinical trials proved that the water extract of *T. terrestris* (WETT) has an antidiabetic activity. The fasting blood glucose, 2-h postprandial glucose, glycosylated haemoglobin and lipid profile of diabetic women treated with TT extract (1000 mg day^−1^) for three months were lowered compared to those of the placebo group [70].

### Prevention and treatment of cardiovascular diseases

Presently, the clinical treatments are thrombolysis and nerve protection. Thrombolysis has a significant effect. However, it is limited by a narrow therapeutic time window. Therefore, the development of neuroprotective agents is of great significance. Studies have shown that GSTT has a neuroprotective effect on cerebral ischaemia injury, and these saponins have been commercially available as active compounds in traditional Chinese medicine formulations, such as “Xin-nao-shutong”, which has been used for the treatment of cardiovascular disease [71]. Meanwhile, TT plays an important role in the treatment of cardiovascular disease with anti-myocardial ischaemia and myocardial ischaemia–reperfusion injury. GSTT has a protective effect on myocardial ischaemia–reperfusion injury. GSTT reduced the levels of lactate dehydrogenase (LDH), methane dicarboxylic aldehyde (MDA), tumor necrosis factor (TNF)-*α* and interleukin (IL)-6, increased SOD and the rate of apoptosis, and improved the structure of cardiomyocytes in rats [72]. Moreover, GSTT could improve coronary flow and heart function and increase adenosine triphosphate (ATP) activity in myocardial ischaemia–reperfusion injury [73]. The methanol extract of *T. terrestris* (METT) fruits, which mainly contains ferulic acid, phloridzin and diosgenin, had an effect on mitochondrial dysfunction in a cell-based (H9c2) myocardial ischaemia model. The extract guards the mitochondria via its antioxidant potential [74]. The cellular and molecular mechanisms of the prevention activity against arthrosclerosis occurs when GSTT significantly suppresses the increase in cell proliferation induced by angiotensin II, significantly suppresses the increase in the intracellular production of hydrogen peroxide (H_2_O_2_) induced by angiotensin II, significantly inhibits the increase in intracellular free Ca^2+^ induced by H_2_O_2_, significantly inhibits the increase in phospho-ERK1/2 induced by angiotensin II, and significantly inhibits the increase in the mRNA expressions of c-fos, c-jun and pkc-*α* induced by angiotensin II [75].

TT significantly suppressed the proliferation of ox-LDL-induced human umbilical vein endothelial cells (HUVECs) and the apoptosis rate. It also prolonged the HUVEC survival time and postponed the cells’ decaying stage (from the 69 h to over 100 h). TT normalized the increased mRNA expressions of PI3K*α* and Socs3. It also decreased the mRNA expressions of Akt1, AMPK*α*1, JAK2, LepR and STAT3 induced by ox-LDL. The most notable changes were for JAK2, LepR, PI3K*α*, Socs3 and STAT3. It is thought that the JAK2/STAT3 and/or PI3K/AKT pathway might be a very important pathway that is involved in the mechanism of TT as a vascular protective agent [76].

In addition, TT functions as a protector of the myocardium. It supported cardioprotective properties against myocardial ischaemia, protected myocardial cells and reduced the apoptosis rate induced by oxidative stress damage [77]. Inhibition of cardiac muscle cell apoptosis occurs when GSTT reduces cell apoptosis through regulating protein expression of Bcl-2 and Bax [78].

### Protective activity in neuronal cells

TT has a protective effect for neuron injury mainly via its anti-inflammatory and antioxidant effects. GSTT has a neuroprotective effect on cerebral ischaemia–reperfusion injury in rats by suppressing NF-κB, TNF-*α* and IL-1*β*. It plays a neuroprotective role in rat cerebral ischaemia reperfusion injury by inhibiting the inflammatory response and PPARγ protein expression [79]. GSTT decreased the damage to PC12 cells induced by H_2_O_2_. The membrane potential of mitochondria and Bcl-2 protein expression in PC12 cells of the GSTT group was significantly increased in a dosage-dependent manner [80]. After cerebral haemorrhaging, brain tissue generates many free radicals that causes lipid peroxidation. GSTT significantly increased the SOD content and decreased the MDA and NO levels in plasma and brain tissue to attenuate neuron injury [81].

The apoptosis of retinal ganglion cells (RGCs) is an important cause of glaucoma. TT can block the optic nerve injury pathway and enhance the survival of the optic nerve to protect the optic nerve [82, 83]. It was reported that TT could reduce the degeneration of RGCs and the retinal nerve fibre layer in hyper-intraocular pressure rabbits by intravenous administration with TT sterilization powder [84].

### Improvement of athletic ability activity

Athletic fatigue is generally measured by the levels of testosterone and corticosterone, and the testosterone and corticosterone (T/C) ratio. Herbs and herbal combinations have been used to improve athletic ability through several ways that mimic epinephrine effects, mimic testosterone effects, and increase the productions of corticotropin and cortisol. TT contains gitonin, protodioscin, and tribulosaponins A and B, which are believed to mimic testosterone-like effects in humans because of the similarities of their chemical structures [85, 86]. The main effect is an increase of testosterone anabolic and androgenic action via the activation of endogenous testosterone production [87].

The administration of GSTT (120 mg kg^−1^) can prolong the time to exhaustion and increase body mass, relative mass, and protein levels of gastrocnemius in overtrained rats. The level of testosterone can directly affect the motor ability of the body and its restoration. Corticosterone can accelerate the decomposition of proteins in the body [88]. Treatment of rats with GSTT during overtraining dramatically increased the serum level of testosterone and led to a significant decrease in the serum level of corticosterone. The T/C ratio with GSTT was much higher than that with the blank control.

In addition, the cognate receptor of testosterone is AR. IGF-1 is closely related to muscle mass, conservation of the musculoskeletal system, the metabolic rate, and muscle strength. GSTT resulted in a significant increase in AR in gastrocnemius and significantly suppressed the overtraining-induced increase in IGF-1R in the liver. It was concluded that GSTT significantly improves exercise performance due to changes in the androgen–AR axis and IGF-1R signalling [89].

#### Antitumour activity

GSTT is likely to affect the processes of apoptosis and metastasis of cancer cells. The overexpression of CXCR4 has been associated with the formation of metastases and poor prognosis of patients with breast and other types of cancer. CCR_7_ is reportedly correlated with lymphatic metastasis and poor prognosis in breast cancers. The product of the BCL2 gene is a mitochondrial membrane protein that blocks apoptosis. After implying a cell-specificity for GSTT, CXCR4 expression was reduced in both cell lines, and CCR_7_ and BCL2 levels decreased only in tumourigenic MCF-7 cells [90].

The anticancer mechanism of terrestrosin D was detected by observing in vitro Caspase-3 activity and vascular endothelial growth factor secretion and the in vivo anticancer effect of the PC-3 xenograft mouse model. It was concluded that terrestrosin D inhibited tumour growth through the inhibition of tumour angiogenesis. In addition, GSTT has a preventive efficacy against UVB-induced carcinogenesis. The photo protective effect of GSTT is tightly correlated with the enhancement of NER gene expression and the blocking of UVB-mediated NF-κB activation [91].

#### Antibacterial activity

The antibacterial activity of TT had been widely studied. A total of 50% of *H. pylori* strains were sensitive to a concentration of 1000 mg mL^−1^ of total extract of TT by the in vitro cup plate method [92]. GSTT inhibited the *Candida albicans* ACS1, ACS2, ERG1, ERG2, ERG6, ERG7, ERG11, ERG25, ERG26 and ERG27 genes, which are directly involved in the ergosterol synthesis pathway. An anti-fungi agent, GSTT may function through direct binding to sterol on the cell membrane and may inhibit ERG gene expression in *C. Albicans* [93]. TT was extracted with different solvents (methanol, petroleum ether, chloroform, and ethanol). The results showed that methanol extract has the highest inhibition zone for *Bacillus cereus*, *Escherichia coli* and *Staphylococcus aureus*. For *Staphylococcus aureus* and *Pseudomonas aeruginosa*, WETT also had a certain inhibitory effect [94]. The ethanol extract of TT exhibited good antibacterial activity against *Streptococcus mutans*, *Streptococcus sanguis*, *Actinomyces viscosus*, *Enterococcus faecalis*, *Staphylococcus aureus*, and *Escherichia coli*. Complexes of *T. terrestris*, *Capsella bursa*-*pastoris*, and *Glycyrrhiza glabra* had synergistic effects compared with those of any of the herbs alone [95].

#### Antioxidant activity

TT exhibited effective antioxidant activity in a concentration-dependent manner by 2,2-di-(4-tert-octylphenol)-1-picrylhydrazyl (DPPH), H_2_O_2_, and superoxide scavenging activity, as well as the FRAP (Ferric reducing antioxidant power) assay [96]. The experiment proved that the antioxidant effect of GSTT is excellent and that it could improve SOD activity and MDA content for chronically high intraocular pressure in rabbits [97]. Compared with the ethanolic extraction, the butanol extract (1 mg ml^−1^) was rich in saponin and had notable quenching of nitric oxide (90.30%), hydroxyl radicals (90.02%), and hydrogen peroxide radicals (89%) [98]. Diosgenin from the callus of *T. terrestris* was found to have great antioxidant activity [99].

#### Anti-inflammatory activity

The EETT and *N*-trans-ρ-caffeoyl tyramine isolated from TT had marked anti-inflammatory activities [100]. EETT and *N*-trans-ρ-caffeoyl tyramine inhibited the productions of nitric oxide (NO), TNF-*α*, IL-6 and IL-10 in lipopolysaccharide (LPS) stimulated RAW264.7 cells in a dose dependent manner. In addition, *N*-trans-ρ-caffeoyl tyramine markedly suppressed the expression of cycloxygense (COX)-2 and the production of prostaglandinE2 (PGE2) through decreasing p-JNK expression.

METT(200 and 400 mg kg^−1^) showed a dose-dependent inhibition of rat paw volume in a carrageenan-induced rat paw edema model. The TT extract and diclofenac sodium (a COX-inhibitor) were injected 30 min prior to carrageenan. The results showed that both drugs can reduce the paw volume 1–4 h after injection of carrageenan by inhibiting the releases of histamine, serotonin and kinins in the early phase. Furthermore, the anti-inflammatory effect of 400 mg kg^−1^ of TT extract is equivalent to that of 20 mg kg^−1^ of Diclofenac sodium [101].

#### Hepatoprotective activity

GSTT can ameliorate injured liver cells and have a protective effect on acute hepatic injury in mice induced by tripterygium glycosides. GSTT can significantly increase the levels of SOD and GPx, decrease the level of MDA in serum, supress Caspase-3 expression and improve the ultrastructure of liver tissue in a mouse model. Caspase-3 is a class of hydrolytic protease, and its activation plays an important role in hepatocyte apoptosis. GSTT can interrupt the cascade in the process of apoptosis by reducing the expression of Caspase-3. The mechanism of its hepatoprotective activity may be related to the antioxidant activity, the influence on metabolism regulation and the repression of apoptosis of liver cells, which effectively reduces the level of Caspase-3 in liver tissue [102].

#### Anthelmintic and larvicidal activity

METT resulted in wormicidal activity by inhibiting spontaneous motility (paralysis) and causing death with lower doses. The effects were comparable with that of Albendazole [103]. In addition, TT exhibited high larvicidal activity. *Anopheles stephensi*, *Aedes aegypti* and *Culex quinque*-*fasciatus* have been identified as the primary vectors of malaria, dengue fever and lymphatic filariasis, respectively, in this part of the desert. The larvicidal potential of TT was evaluated by calculating the mortality percent of *A. stephensi*, *A. aegypti* and *C. quinque*-*fasciatus*. The results showed that the fruits were a more potent form regarding larvicidal activity than the leaves [104].

#### Anticarious activity

TT was certified to have an anticarious effect. *Streptococcus mutans* is an important oral pathogen that causes dental caries. The anticarious effect of TT was evaluated for inhibiting *S. mutans* bacteria. *In vitro* studies showed that the extract exhibited antibacterial activity for inhibiting *S. mutans* growth in a dose dependent manner. Meanwhile, TT extract suppressed the adherence of *S. mutans* to saliva-coated hydroxyapatite (S-HA), which simulated teeth, and inhibited the formation of water-insoluble glucans [105].

### Antiaging and memory improvement activity

GSTT can effectively increase SOD activity, decrease MDA and hydroxyproline (Hyp) in the skin and increase the activities of CAT and GPx in the whole blood of d-galactose-induced senile mice. Compared with the ageing model group, the GSTT group showed a thicker dermis and more compactly arranged fibre content. The skin morphology of the GSTT group was close to that of the normal group [106].

Ageing is accompanied by a decline in memory, but GSTT can improve memory impairment. A study showed that GSTT significantly improved obtained memory disorder, consolidated memory disorder and recovered memory disorder [107]. The effect of the water extract of TT fruits on learning and memory ability in rodents was evaluated by recording the time of reaching the reward chamber (TRC) in the Hebb William Maze and the transfer latency (TL) in the T-zema. The results showed that the water extract of TT fruits significantly reduced the time of arrival at the maze in a dose-dependent manner [108].

#### Absorption enhancer

TT promotes absorption. The biopharmaceutics classification system (BCS) is a scientific classification method based on solubility in vitro and permeability of drugs in the intestine. Metformin hydrochloride (HCl) is a BCS class III drug with a high solubility and poor absorption characteristics. Therefore, it is necessary to increase the intestinal permeability of drugs to improve their bioavailability. The experiment indicated that TT can enhance the absorption of Metformin HCl in a goat intestine [109]. The absorption enhancement effect of TT was concluded by the presence of saponin.

### Toxicity

An animal study investigated the acute toxicity of METT (2 g kg^−1^, given orally to 5 mice for 14 days). The methanol extracts mainly consisted of flavonoids, anthraquinones, phenols/tannins, and steroids/triterpenes. As a result, there were no toxic symptoms or mortality observed in any animals and no obvious differences between the treated and control animals regarding behavioural changes and toxicological signs (general behaviour, motor activities, aggressiveness, reaction to noise, reaction to pinch, state of the tail and state of excrement) [110].

The genotoxic potential of TT extracts, as assessed by a Comet assay in a rat kidney cell line and by an Ames assay in *Salmonella typhimurium* strains, was evaluated [111]. The METT had relatively higher genotoxic activities (2400 mg mL^−1^ METT, tDNA%: 11.43) and cytotoxic activities (IC_50_ = 160 mg mL^−1^) than WETT and the chloroform extracts of *T. terrestris* (CETT) but did not damage the deoxyribonucleic acid (DNA), whereas the 300 mg mL^−1^ WETT might induce frame shift mutations when metabolically activated. WETT showed oestrogenic activity at concentrations higher than 27 mg mL^−1^ (2.6-fold), and none of the extracts had androgenic activity.

## Conclusions

The traditional pharmacological activities of TT focused on improving sexual function and cardiotonic properties. Modern investigation showed that steroidal saponins and flavonoids with the prominent antiaging and anti-inflammatory activities were the main contributors to the traditional pharmacological activities. While the clinical trials with TT are scarce, and randomized placebo controlled clinical trials should be done in future. In addition, we should give more attention to the traditional curative effect of skin pruritus. TT maybe have more utilization as cosmetic plant materials on skin. A critical assessment of the results presented in this review may provide scientific evidence for the reasonable utilization of TT and promote further investigation for the development of new herbal medicine and health products.

## Abbreviations

ATP: adenosine triphosphate
CaOx: calcium oxalate
CAT: catalase
CCD_7_: carotenoid cleavage dioxygenase 7
CETT: chloroform extracts of *T. terrestris*
CO_2_: carbon dioxide
COD: calcium oxalate dihydrate
COM: calcium oxalate monohydrate stones
COX: cycloxygense
DHEAS: dehydroepiandrosterone sulphate
DHT: dihydrotestosterone
DNA: deoxyribonucleic acid
DPPH: 2,2-di-(4-tert-octylphenol)-1-picrylhydrazyl
ED: erectile dysfunction
EETT: ethanol extract of *T. terrestris*
EL: ejaculation latency
FRAP: ferric reducing antioxidant power
GPx-1: glutathione peroxidase 1
GSTT: gross saponins of *T. terrestris*
HCl: hydrochloride
H_2_O_2_: hydrogen peroxide
HSDD: hypoactive sexual desire disorder
HUVECs: human umbilical vein endothelial cells
Hyp: hydroxyproline
IIEF: International Index of Erectile Function
IF: intromission frequency
IL: interleukin
LDH: lactate dehydrogenase
LDL: low density lipoprotein
LPS: lipopolysaccharide
MDA: methane dicarboxylic aldehyde
METT: methanolic extract of *T. terrestris*
MF: mounting frequency
ML: mounting latency
MTZ: metronidazole
NO: nitric oxide
PEI: post-ejaculation interval
PGE2: prostaglandin E2
RGCs: retinal ganglion cells
S-HA: saliva-coated hydroxyapatite
SOD: superoxide dismutase
T: testosterone
T/C: testosterone and corticosterone
TL: transfer latency
TNF: tumor necrosis factor
TRC: reward chamber
TT: *Tribulus terrestris* L.
TT-FG: the furostenol glycoside fraction of *T. terrestris* extract
WETT: water extract of *T. Terrestris*

## References

[1] Chhatre S, Nesari T, Somani G, Kanchan D, Sathaye S (2014). Phytopharmacological overview of *Tribulus terrestris*. Pharmacogn Rev.

[2] Neychev V, Mitev V (2016). Pro-sexual and androgen enhancing effects of *Tribulus terrestris L.*: fact or fiction. J Ethnopharmacol.

[3] Shang ZJ (2008). Annotation of Shen Nong Ben CaoJ ing.

[4] Chinese Pharmacopoeia Commission (2015). Chinese pharmacopoeia (volume I).

[5] Mohammed MS, Khalid HS, Osman WJA, Muddathir AK (2014). A review on phytochemical profile and biological activites of three anti-inflammatory plants used in sudanese folkloric medicine. Am J Pharm Tech Res.

[6] Akram M, Asif HM, Akhtar N, Shah PA, Uzair M, Shaheen G (2011). *Tribulus terrestris Linn.*: a review article. J Med Plants Res.

[7] Xu YX, Chen HS, Liu WY, Gu ZB, Liang HQ (1998). Two sapogenins from *Tribulus terrestris*. Phytochemistry.

[8] Mahato SB, Sahu NP, Ganguiy AN, Kazumoto M, Toshio K (1981). Steroidal glycosides of *Tribulus terrestris Linn*. J Chem Soc Perkin I.

[9] Xu YX, Chen HS, Liang HQ, Gu ZB, Liu WY, Leung WN (2000). Three new saponins from *Tribulus terrestris*. Planta Med.

[10] Tomova M, Panova D, Wulfson NS (1974). Steroid saponins and sapogenins IV. Saponins from *Tribulus terrestris*. Planta Med.

[11] Wang Y, Othani K, Kasai R, Yamasaki K (1996). Steroidal saponins from fruits of *Tribulus terrestris*. Phytochemistry.

[12] Huang JW, Tan CH, Jiang SH, Zhu DY (2003). Terrestrinins A and B, two new steroid saponins from *Tribulus terrestris*. J Assian Nat Prod Res.

[13] Deepak M, Dipankar G, Prashanth D, Asha MK, Amit A, Venkataraman BV (2002). Tribulosin and b-sitosterol-dglucoside.the anthelmintic principles of *Tribulus terrestris*. Phytomedicine.

[14] De Kock WT, Enslin PR (1958). Chemical investigation of photosensitization diseases of domestric animals I. Isolation andcharacterization of steroidal sapogenins from *Tribulus terrestris*. Afr Chem Inst.

[15] Sharma HC, Narula JL (1977). Chemical investigation of flowers of *Tribulus terrestris*. Chem Era.

[16] Wang Y, Othtani K, Kasai R, Yamasaki K (1997). Steroidal saponins from fruits of *Tribulus terrestris*. Phytochemistry.

[17] Huang JW, Jiang SH, Tan CH, Zhu DY (2002). Structural elucidation of three new steroid sapogenins. Chin J Org Chem.

[18] Wu G, Jiang S, Jiang F, Zhu D, Wu H, Jiang S (1996). Steroidal glycosides from *Tribulus terrestris*. Phytochemistry.

[19] Xu YJ, Xie SX, Zhao HF, Han D, Xu TH, Xu DM (2001). Studies on the chemical constituents from *Tribulus terrestris*. Acta Pharm Sin.

[20] Cai LF, Wu YJ, Zhang JG, Pei FK, Xu YJ, Xie SX (2001). Steroidal saponins from *Tribulus terrestris*. Planta Med.

[21] Bedir E, Khart IA, Walker LA (2002). Biologically active steroidal glycosides from *Tribulus terrestris*. Pharmazie.

[22] Gheorghiu A, Ionescu-Matiu E (1968). Presence of chlorogenin next to diosgenin and gitogenin in *Tribulus terrestris*. Ann Pharm.

[23] Wang ZF, Wang BB, Zhao Y, Wan FX, Sun Y, Guo RJ (2016). Furostanol and spirostanol saponins from *Tribulus terrestris*. Molecules.

[24] Kang LP, Wu KL, Yu HS, Pang X, Liu J, Han LF (2014). Steroidal saponins from *Tribulus terrestris*. Phytochemistry.

[25] Lan S, Gang C, Feng SG, Wei W, Li ZF, Chen H (2009). Steroidal saponins from *Tribulus terrestris*. Steroids.

[26] Su L, Feng SG, Qiao L, Zhou YZ, Yang RP, Pei YH (2010). Two new steroidal saponins from *Tribulus terrestris*. Chin Chem Lett.

[27] Matschenko HE, Gulemetova R, Kintya PK, Shashkov AS (1990). A sulfated glycoside from the preparation“Tribestan”. Khim Prir Soedin.

[28] Conrad J, Dinchev D, Klaiber I, Mika S, Kostova I, Kraus W (2004). A novel furostanol saponin from *Tribulus terrestris* of Bulgarian origin. Fitoterapia.

[29] Wilkins AL, Miles CO, De Kock WT, Erasmus GL, Basson AT, Kellerman TS (1996). Photosensitivity in South Agrica. IX. Structure elucidation of a beta-glucosidease-treated saponin from T-6lflus terrestris, and the identification of saponin ehemotypes of South Agrican *T. terrestris*. Onderstepoort J Vet Res.

[30] Sun WJ, Gao J, Tu GZ, Guo Z, Zhang Y (2002). A new steroidal saponin from *Tribulus terrestris Linn*. Nat Prod Lett.

[31] Cai LF, Jing FY, Zhang JG, Pei FK, Xu YJ, Liu SY (1999). Steroidal saponins from *Tribulus terrestris*. Acta Pharm Sin.

[32] Kostova I, Dinchev D, Rentsch GH, Dimitrov V, Lvanova A (2002). Two new sulfated furostanol saponins from *Tribulus terrestris*. Z Naturforsch.

[33] Liu T, Lu X, Wu B, Chen G, Hua HM, Pei YH (2010). Two new steroidal saponins from *Tribulus terrestris L*. J Asian Nat Prod Res.

[34] Qu NN, Yang SS (2007). Extraction and determination of chemical constituents of flavonides in *Tribulus terrestris L*. J Liaoning Univ Tradit Chin Med.

[35] Saleh NAM, Ahmed AA, Abdalla MF (1982). Flavonoid glycosides of *Tribulus pentandrus* and *T. Terrestris*. Phytochemistry.

[36] Marzieh M, Yekta S (2008). Flavonoid Glycosides from *Tribulus terrestris L.* orientalis. Iran. J Pharm Sci.

[37] Yang FK, Zhang ML, Quan XL, Xue ML, Cai HX, Yang J (2014). Determination and comparison HPLC of total flavonoids in different parts of *Tribulus Terrestris* glycoside content. Mod Tradit Chin Med.

[38] Su L, Feng SG, Qiao L, Zhou YZ, Yang RP, Pei YH (2009). Two new steroidal saponins from *Tribulus terrestris*. J Asian Nat Prod Res.

[39] Bhutani SP, Chibber SS, Seshadri TR (1969). Flavonoids of the fruits and leaves of *Tribulus terrestris*: constitution of tribuloside. Phytochemistry.

[40] Zhang XP, Wei N, Huang J, Tan YF, Jin DJ (2012). A new feruloyl amide derivative from the fruits of *Tribulus terrestris*. Nat Prod Res.

[41] Wu TS, Shi LS, Kuo SC (1999). Alkaloids and other constituents from *Tribulus terrestris*. Phytochemistry.

[42] Lv AL (2007). Chemical constituents of *Tribulus terrestris L*.

[43] Wang Y (1989). Pharmacological action and chemical components of *Tribulus terrestris* L. (A Review). J Beijing Univ Trad Chin Med.

[44] Lv AL, Zhang N, Sun MG, Huang YF, Sun Y, Ma HY (2008). One new cinnamic imide derivative from the fruits of *Tribulus terrestri*s. Nat Prod Res.

[45] Ren YJ, Chen HS, Yang JY, Zhu H (1994). Isolation and identification of a new derivative of cinnamic amide from *Tribulus Terrestris*. Acta Pharm Sin.

[46] Wang RY, Chen G, Yu CY (2009). Chemical constituents of *Tribulus terrestris L*. J B Univ Chem Technol (Nat Sci Ed).

[47] Li RH (2006) Studies on bioactive compounds and quality evaluation of *Tribulus terrestris L*. J Liaoning Univ Tradit Chin Med

[48] Chen HS, Chen QJ, Xuan WD (2004). A new organic acid from *Tribulus terrestris*. Acad J Second Military Med Univ.

[49] Wang XD, Shao JX (1994). Determination of amino acids in *Tribulus terrestris L*. Amino Acid Biotic Resour.

[50] Lv AL, Zhang Y, Ma HY, Wang D, Dang Q, Pei YH (2007). Chemical constituents of *Tribulus terrestris L*. Chin J Med Chem.

[51] Liu J, Chen HS, Xu YX, Zhang WD, Liu WY (2003). Studies on chemical constituents of *Tribulus terrestris L*. Acad J Second Military Med Univ.

[52] Custers D, Van PN, Courselle P, Apers S, Deconinck E (2017). Chromatographic fingerprinting as a strategy to identify regulated plants in illegal herbal supplements. Talanta.

[53] Sahin K, Orhan C, Akdemir F, Tuzcu M, Gencoglu H, Sahin N (2016). Comparative evaluation of the sexual functions and NF-κB and Nrf2 pathways of some aphrodisiac herbal extracts in male rats. BMC Complement Altern Med.

[54] Kalamegam G, Adaikan PG (2008). The hormonal effects of *Tribulus terrestris* and its role in the management of male erectile dysfunction—an evaluation using primates, rabbit and rat. Phytomedicine.

[55] Tyagi RM, Aswar UM, Mohan V, Bodhankar SL, Zambare GN, Thakurdesai PA (2008). Study of furostenol glycoside fraction of *Tribulus terrestris* on male sexual function in rats. Pharm Biol.

[56] Kamenov Z, Fileva S, Kalinov K (2017). Evaluation of the efficacy and safety of *Tribulus terrestris*, in male sexual dysfunction—a prospective, randomized, double blinded, placebo-controlled clinical trial. Maturitas.

[57] Oliveira NNPM, Félix MAR, Pereira TCS, Rocha LGP, Miranda JR, Zangeronimo MG (2015). Sperm quality and testicular histomorphometry of wistar rats supplemented with extract and fractions of fruit of *Tribulus terrestris L*. Brazarch Biol Techn.

[58] Kumari M, Singh P (2015). Protective role of *Tribulus terrestris* on aluminium chloride-induced reproductive toxicity in the male laboratory mouse. Int J Pharm Pharm Sci.

[59] Kumar P, Singh P (2015). *Tribulus terrestris* ameliorates metronidazole-induced spermatogenic inhibition and testicular oxidative stress in the laboratory mouse. Iindian J Pharmacol.

[60] Khaleghi S, Bakhtiari M, Asadmobini A, Esmaeili F (2016). *Tribulus terrestris* extract improves human sperm parameters in vitro. J Evid Based Complement Altern Med.

[61] Bitzer J, Giraldi A, Pfaus J (2013). Sexual desire and hypoactive sexual desire disorder in women. introduction and overview. standard operating procedure. J Sexual Med.

[62] De Souza KZD, Vale FBC, Geber S (2016). Efficacy of *Tribulus terrestris* for the treatment of hypoactive sexual desire disorder in postmenopausal women: a randomized, double-blinded, placebo-controlled trial. Menopause.

[63] Postigo S, Lima SMRR, Yamada SS, Reis BFD, Silva GMDD, Aoki T (2016). Assessment of the effects of *Tribulus terrestris* on sexual function of menopausal women. Rev Bras Ginecol Obstet.

[64] Sharma I, Khan W, Ahmad S (2017). *In vitro* and ex vivo approach for anti-urolithiatic potential of bioactive fractions of gokhru with simultaneous HPLC analysis of six major metabolites and their exploration in rat plasma. Pharm Biol.

[65] Aggarwal A, Tandon S, Singla SK, Tandon (2012). A novel antilithiatic protein from *Tribulus terrestris* having cytoprotective potency. Protein Peptide Lett.

[66] Arasaratnam V, Balakumar S, Senthuran A, Rajendraprasad R (2010). A study of *Tribulus terrestris* extract on risk factors for urinary stone in normal subjects and urolithic patients. J Natn Sci Found Sri Lanka.

[67] Van FA, Lucassen PL, Akkermans RP, Van de Lisdonk EH, Rutten GE, Van Weel C (2006). *α*-Glucosidase inhibitors for type 2 diabete smellitus (Review). Cochrane DB Syst Rev.

[68] Ercan P, El SN (2016). Inhibitory effects of chickpea and *Tribulus terrestris* on lipase, *α*-amylase and *α*-glucosidase. Food Cherm.

[69] Zhang SJ, Feng SC (2012). Effects of saponins from *Tribulus terrestris* on postprandial blood glucose levels in normal and type 2 diabetic rats. Pract Pharm Clin Remed.

[70] Samani NB, Jokar Soveid M, Heydari M, Mosavat SH (2006). Efficacy of *Tribulus terrestris* extract on the serum glucose and lipids of women with diabetes mellitus. Iran J Pharm Sci.

[71] Zhang S, Li H, Xu H, Yang SJ (2010). Effect of gross saponins of *Tribulus terrestris* on cardiocytes impaired by adriamycin. Acta Pharm Sin.

[72] Li Y, Song HY, Zhang YG, Yu FL, Zhang S, Li H (2010). Protection of gross saponins of *Tribulus terrestris* (GSTT) on cardiac ischemia-reperfusion injury. Chin Tradit Herbal Drugs.

[73] Zhang S, Li H, Wei ZR, Liang L, Zhang W, Yang SJ (2010). Influence of gross saponins from *Tribulus terrestris* preconditioning on myocardial ischemia-reperfusion injury. Chin Tradit Herbal Drugs.

[74] Reshma PL, Sainu NS, Mathew AK, Raghu KG (2016). Mitochondrial dysfunction in H9c2 cells during ischemia and amelioration with *Tribulus terrestris L*. Life Sci.

[75] Li M, Guan Y, Liu J, Zhai F, Zhang X, Guan L (2013). Cellular and molecular mechanisms in vascular smooth muscle cells by which total saponin extracted from *Tribulus terrestris* protects against artherosclerosis. Cell Physiol Biochem.

[76] Jiang YH, Yang CH, Li W, Wu S, Meng XQ, Li DN (2016). Aqueous extracts of *Tribulus terrestris* protects against oxidized low-density lipoprotein-induced endothelial dysfunction. Chin J Integr Tradit West Med.

[77] Reshma PL, Lekshmi VS, Sankar V, Raghu KG (2015). *Tribulus terrestris (Linn.)* Attenuates Cellular Alterations Induced by Ischemia in H9c2 Cells Via Antioxidant Potential. Phytother Res.

[78] Guo Y, Yin HJ, Shi DZ (2006). Effect of xinnao shutong capsule on cardiac muscle cell apoptosis and protein expressions of bcl-2 and bax in hyperlipidemia rats after myocardial infarction. Chin J Integr Tradit West Med.

[79] Fg Zhai, Li HZ, Zhou FB, Lin F, Guan LX (2015). Effects of saponins of *Tribulus terrestris* on PPARγ and NF-κB signaling pathways expression in rat brain following cerebral ischemic injury. Med Recapitulate.

[80] Jiang EP, Su XJ, Li H, Yang SJ (2008). Protective effects and mechanisms of gross saponins of *Tribulus terrestris* on apoptosis of PC12 cells induced by H_2_O_2_. Chin Tradit Herbal Drugs.

[81] Li LB, Li J, Li H (2006). Protective effects of gross saponins of *Tribulus terrestris* on experimental intracerebral hemorrhage in rats. J Harbin Med Univ.

[82] Wang ZJ (2011). Progress on protective mechanisms of single Chinese crude drug and its effective ingredients on optic nerve of glaucosis. Herald Med.

[83] Sheng YM, Meng XL (2007). Research advance on the effect of several Chinese traditional medicine on optic nerve protection. Herald Med.

[84] Liao MY, Huang LN, Zeng P (2009). Effect of *Tribulus terrestris L.* on the retinal ganglion cells. Int Eye Sci.

[85] Bucci LR (2000). Selected herbals and human exercise performance. Am J Clin Nutr.

[86] Di PM (1995). Anabolic steroids substitutes from plants and herbs?. Drugs Sports.

[87] Saudan C, Baume N, Emery C (2008). Short term impact of *Tribulus terrestris* intake on doping control analysis of endogenous steroids. Forensic Sci Int.

[88] Jing QG (1998). The effect of overload-training on the pituitary-gonadal axis of rats. Zhejiang Sport Sci.

[89] Yin L, Wang Q, Wang X, Song LN (2016). Effects of *Tribulus terrestris* saponins on exercise performance in overtraining rats and the underlying mechanisms. Can J Physiol Pharmacol.

[90] Goranova TE, Bozhanov SS, Lozanov VS, Mitev VI, Kaneva RP, Georgieva EI (2015). Changes in gene expression of CXCR_4_, CCR_7_ and BCL_2_ after treatment of breast cancer cells with saponin extract from *Tribulus terrestris*. Neoplasma.

[91] Sisto M, Lisi S, D’Amore M, De Lucro R, Carati D, Castellana D (2012). Saponins from *Tribulus terrestris L.* protect human keratinocytes from UVB-induced damage. J Photochem Photobiol B: Biol.

[92] Vala MH, Goudarzi H, Moghada SN, Nejad MK, Jahangiti S, Gholami M (2013). *In vitro* assessment of *Tribulus terrestris* aqueous extract and Benzoxacin fraction against *Helicobacter pylori* isolates from biopsy samples of Iranian patients. Novel biomed.

[93] Zhang JD, Zheng XU, Cao YB, Jun GU, Jiang YY (2011). Study on regulation of ERG genes expression in Candida albicans by a new anti-fungi agent TTS-12. Chin Pharm J.

[94] Kiran B, Lalitha V, Raveesha KA (2011). *In vitro* evaluation of aqueous and solvent extract of *Tribulus terrestris L.* leaf against human bacteria. Int J Pharm Tech Res.

[95] Soleimanpour S, Sedighinia FS, Safipour AA, Zarif R, Ghazvini K (2015). Antibacterial activity of *Tribulus terrestris* and its synergistic effect with *Capsella bursa*-*pastoris* and *Glycyrrhiza glabra* against oral pathogens: an in vitro study. Avicenna J Phytomed.

[96] Bhuvad S, Nishteswar K (2016). Assessment of free radical scavenging activity of ten madhuraskandha drugs through UV spectroscopic and chromatographic technique. J Pharm Pharm Sci.

[97] Li N, Huang LN, Zeng P (2013). Influence of gross saponins from *Tribulus terrestris L.* on SOD activity and MDA content for chronic high intraocular pressure in rabbit. Int Eye Sci.

[98] Hemalatha S, Hari R (2013). Comparative antioxidant activities of crude ethanolic and saponin rich butanol extracts of *Tribulus terrestris* fruits. Int J Pharma Biol Sc.

[99] Yogendra KG, Vimala Y, Yogendra KG (2014). Antioxidant activity and RP-HPLC analysis of diosgenin from the callus of *Tribulus terrestris Linn*. Int J Res Ayurveda Pharm.

[100] Ko HJ, Ahn EK, Oh JS (2015). *N*-trans-*ρ*-caffeoyl tyramine isolated from *Tribulus terrestris* exerts anti-inflammatory effects in lipopolysaccharide-stimulated RAW 264.7 cells. Int J Med Microbiol.

[101] Baburao B, Rajyalakshmi G, Venkatesham A, Kiran G, Shyam Sunder A, Ganga Rao B (2009). Anti-inflammatory and antimicrobial activities of methanolic extract of *Tribulus terrestris Linn* plant. Int J Chem Sci.

[102] Hu DH (2009). Effect of gross saponins from *Tribulus terrestris* on hepatic apoptosis in mice’s acute hepatic injury induced by tripterygium glycosides.

[103] Parimala DR (2011). *In*-*vitro* anthelmintic activity of methanolic extract of aerial parts of *Tribulus terrestris Linn*. J Global Pharma Technol.

[104] Bansal SK, Singh KV, Sharma S (2014). Larvicidal potential of wild mustard (*Cleome viscosa*) and gokhru (*Tribulus terrestris*) against mosquito vectors in the semi-arid region of Western Rajasthan. J Environ Biol.

[105] Oh HK, Park SJ, Moon HD, Jun SH, Choi NY, You YO (2011). *Tribulus terrestris* inhibits caries-inducing properties of *Streptococcus mutans*. J Med Plants Res.

[106] Zhu XW (2011). Effect of gross saponins from *Tribulus terrestris* on skin morphology of d-galactose-induced aging mice. Chin J Gerontol.

[107] Zhang J, Zhang DS, Yan CL (2007). Effects of GSTT on dysmnesia in mice. Pharmacol Clin Chin Mater Clin Med.

[108] Prabhu N (2014). Effect of *Tribulus terrestris* on learning and memory in wistar rats. Phcog J.

[109] Ayyanna C, Chandra Mohan Rao G, Sasikala M, Somasekhar P, Arun Kumar N, Pradeep Kumar MVS (2012). Absorption enhancement studies of Metformin hydrochloride by using *Tribulus terrestris* plant extract. Int J Pharm Tech.

[110] EI-Shaibany A, Al-Habori M, Al-Tahami B (2015). Anti-hyperglycaemic activity of *Tribulusterrestris L.* aerial part extract in glucoseloaded normal rabbits. Trop J Pharm Res.

[111] Abudayyak M, Jannuzzi AT, Özhan G, Alpertunga B (2015). Investigation on the toxic potential of *Tribulus terrestris in vitro*. Pharm Biol.

